# Village Response to Mass Drug Administration for Schistosomiasis in Mwanza Region, Northwestern Tanzania: Are We Missing Socioeconomic, Cultural, and Political Dimensions?

**DOI:** 10.4269/ajtmh.19-0843

**Published:** 2020-09-08

**Authors:** Joseph R. Mwanga, Safari M. Kinung’hi, Justina Mosha, Teckla Angelo, Jane Maganga, Carl H. Campbell

**Affiliations:** 1 Department of Epidemiology, Biostatistics and Behavioral Sciences, School of Public Health, Catholic University of Health and Allied Sciences, Mwanza, Tanzania;; 2 National Institute of Medical Research (NIMR), Mwanza Center, Mwanza, Tanzania;; 3 Schistosomiasis Consortium for Operational Research and Evaluation (SCORE), Center for Tropical and Emerging Global Diseases (CTEGD), University of Georgia, Athens, Georgia

## Abstract

Praziquantel (PZQ)-based mass drug administration (MDA) is the main approach for controlling schistosomiasis in endemic areas. Interventions such as provision and use of clean and safe water, minimizing contacts with infested water, disposal of human waste in latrines, and snail control provide additional key interventions to break the transmission cycle and could complement and perhaps sustain the benefits of MDA. However, all interventions deployed need to be accepted by the targeted communities. A qualitative study was conducted to examine factors that might differentiate villages which did not show a substantial decrease in *Schistosoma mansoni* prevalence despite repeated, high treatment coverage referred to as “persistent hotspot (PHS) villages” from villages which showed a substantial decrease in prevalence referred to as “responding (RES) villages.” A convenient sample of adults was drawn from eight villages. Thirty-nine key informants were interviewed and 16 focus groups were held with a total of 123 participants. Data were analyzed manually using a thematic content approach. In both PHS and RES villages, schistosomiasis was not considered to be a priority health problem because of its chronic nature, lack of knowledge and awareness, and poverty among study communities. Persistent hotspot villages exhibited poor leadership style, lack of or insufficient social engagement*,* little or lack of genuine community participation, little motivation, and commitment to schistosomiasis control compared with RES villages where there were commitment and motivation to fight schistosomiasis. We support the view of scholars who advocate for the adoption of a biosocial approach for effective and sustainable PZQ-based MDA for schistosomiasis control.

## INTRODUCTION

Human schistosomiasis is also known as bilharziasis. It is a chronic water-related parasitic disease, caused by infection with blood flukes (trematode worms) of the genus *Schistosoma.* It is one of the world’s most prevalent neglected tropical diseases in the 78 tropical and subtropical countries, of which 42 are in sub-Saharan Africa.

*Schistosoma mansoni* and *Schistosoma haematobium* are the most prevalent schistosome species in sub-Saharan Africa. Schistosomiasis poses a significant health threat to almost 800 million people globally, with about 200 million people estimated to be infected.^1,2^ More than 90% of all *Schistosoma* infections are found in sub-Saharan Africa,^3^ and Tanzania is the second country after Nigeria for harboring the highest numbers of cases of schistosomiasis.^4^ Schistosomiasis can present as an acute illness, but its main public health impact is due to chronic infections.^5^ It has a greater than generally perceived impact on human health in terms of the yearly disease burden, mortality, and loss of economic productivity (i.e., disability-adjusted life years) associated with the disease.^6,7^ Schistosomiasis is highly endemic in Mwanza region in northwestern Tanzania, especially among communities located on the shores of Lake Victoria.^8–11^

In the absence of a safe and effective vaccine, the WHO has recommended a schistosomiasis control strategy known as preventive chemotherapy (PC), where at-risk populations should receive regular (usually annual) single doses of praziquantel (PZQ), regardless of their infection status. Praziquantel is currently the drug of choice for preventive treatment of schistosomiasis because it is effective against all *Schistosome* species, can be administered as a single oral dose, is affordable, and relatively safe.^12^ Periodic PZQ-based mass drug administration (MDA) of communities is the most widely used approach to control schistosomiasis in endemic communities^13^ with varying levels of success.^14,15^ Preventive chemotherapy–based schistosomiasis control programs have been implemented in several African countries including Tanzania. The current schistosomiasis control strategy in Tanzania focuses on MDA with PZQ in school-age children and started in 2004. The northwestern regions of Tanzania, around Lake Victoria, have been the major focus for MDA to control intestinal schistosomiasis in school-age children.

Besides, as this study shows, successful disease control also needs to take into consideration sociocultural factors along with other interventions implemented. Although PC at high coverage (≥ 75%) can contribute to the eventual control of schistosomiasis, there are specific cultural, social, economic, and political factors that need to be considered to obtain acceptance of the interventions and ultimate transmission reduction. These factors give insights into the way people seek health care, accept and adopt interventions such as PC, and cooperate with researchers or other healthcare providers. Therefore, an understanding of the social, political, cultural, and economic factors surrounding human behavior is crucial for implementing effective control interventions. New tools that incorporate social science approaches and strategies to complement current interventions for schistosomiasis control and eventual elimination of schistosomiasis are urgently required.^16^ Schistosomiasis is widely recognized as a disease that is socially determined.^16,17^

Thus, social science research using qualitative methods can offer insights into the dynamics of schistosomiasis transmission and control that can complement biomedical interventions.^18^ The importance of social, economic, and cultural perspectives to further our understanding of the epidemiology and control of schistosomiasis in Africa has been underscored elsewhere.^16,17^ Because human behavior plays such an important role in the transmission of schistosomiasis, any control programs must take into account this human element.^19^ Hence, in this article, we underscore the importance of understanding the social context of PZQ-based MDA.

In 2010, the Schistosomiasis Consortium for Operational Research and Evaluation (SCORE) launched large-scale studies in Tanzania and other African countries aimed at determining optimal strategies for MDA that provide the greatest reductions in the prevalence and intensity of schistosomiasis infections in school-aged children following 4 years of MDA. In Mwanza, northwestern Tanzania, 150 villages were involved.

However, after four rounds of MDA with high coverage, still half of the villages remained at high prevalence of *S. mansoni* (28–73%) compared with another half which showed a substantial decline in prevalence (1–16%) over the same period. Against such a background, a study was conducted with the overall objective of examining factors that might differentiate villages which did not show a substantial decrease in *S. mansoni* prevalence despite repeated, high coverage MDA, herein referred to as persistent hotspots (PHS), from villages which showed a substantial decrease in prevalence referred to as responding (RES) villages.

In this article, we focus on the identification of factors that contributed to the ongoing high *S. mansoni* prevalence found in these PHS villages. We hypothesized that there were socioeconomic, cultural, and political factors that might have been responsible for the failure to realize the decrease in prevalence for these PHS villages that were seen in the RES villages. Specifically, the focus was on the following study variables: perceptions on common illnesses in the study villages, village structure, socioeconomic status, villages, and school leadership quality; migration and people’s movements; community engagement; motivation; and commitment to schistosomiasis control interventions. In this article, we use action and action competence (AC) perspectives^20^ to explore factors that might differentiate villages that did not show substantial decrease in *S. mansoni* prevalence despite repeated, high treatment coverage (i.e., PHS) from villages that responded to MDA by showing a substantial decrease in prevalence (i.e., RES). We also make use of the health promotion approach which recognizes the need to support health education through building healthy public policy and creating supportive environments for health. The social determinants of health framework is also applied.

In this study, we consider action as something that is performed consciously and that it has been considered and is targeted. This then distinguishes it from concepts such as activity, movements, and habits by the deliberateness of the action.^21^ Actions are therefore intentional and must be focused on the resolution of a problem which the actor sees as important, and has to be addressed to cause rather than symptoms.

The health promotion perspective in this article is based on the AC approach. Embedded in liberal education theory, AC is broadly defined as “formative ideal in democratic perspective.”^22^ Schnack defines AC as: “capability based on critical thinking and incomplete knowledge to involve yourself as a person with other persons in responsible actions and counteractions for a more humane world.”^22^ Action competence consists of several components; first, insights and knowledge: a broad, positive, coherent, and action-minded understanding of health problems of concern; second, commitment: motivation to become involved in changes regarding one’s own life in the society; third, vision: the ability to go behind the health issue and think creatively and critically; and fourth, action experience or real experience from participating individually or collectively in health-promoting changes.^23^

The AC approach has been operationalized in the form of the so-called Investigation, Vision, Action, and Change (IVAC) model, which stands for 1) investigation of the health issue, 2) developing visions, and 3) taking action, and 4) facilitating change.^24^ Rather than being viewed as steps or phases in a certain order, the four elements in IVAC-model are suggested as perspectives that constitute a mental framework and steering tool in action-oriented and health promotion direction. In brief, AC is a social process of identifying causes of problems (in this case what causes *S. mansoni*) and the condition that needs to be changed (in this case high prevalence of *S. mansoni* in PHS villages), and finally explores action possibilities (what actions are possible) and selects what are considered to be appropriate actions.^23^

## MATERIALS AND METHODS

### Study area and population.

The study was conducted in Mwanza region, northwestern Tanzania. Mwanza region is located to the south of Lake Victoria between latitude 1°30′ and 3°2′ south of the equator and longitude 31°45′ and 34°10′ east of the Greenwich meridian.

The study population was predominantly Wasukuma ethnic group who live in parts of Sukumaland, which is located to the west and south of Lake Victoria. The economy of Mwanza region is dominated by several major activities, but the most important is agriculture, and the region is one of the largest producers of cotton, which is also a major cash crop. Major food crops produced are paddy, maize, cassava, sorghum, millet, and sweet potatoes. Livestock-keeping (cattle, goats, sheep, and chicken) is another economic activity carried out in Mwanza region always linked with crop production within the framework of individual households. Fishing is yet another economic activity for some people of Mwanza region, especially those living close to the lake shores and those living in numerous islands of Lake Victoria. Thus, land, livestock, and Lake Victoria waters constitute the main components of the Wasukuma economic system, the basis of which is agriculture founded on family labor.

### Study design and sampling.

The study was a cross-sectional descriptive qualitative inquiry that involved studying eight villages to describe and compare the socioeconomic, political, and cultural practices related to schistosomiasis infection. The study participants were conveniently selected from among adult key informants (four PHS villages), namely, Bulima, Shinembo, Luchili, and Karumo, and another four from RES villages, namely, Kahumulo, Igalagalilo, Shilingwa, and Mhonze. The key informants (KIs) comprised village leaders, primary school teachers, religious leaders, community health workers, and adult male and female villagers. Village leaders and school head teachers facilitated the availability of adult key informants in primary school premises and the villages, respectively.

### Methods for data collection.

Key informants’ interviews (KIIs) and focus group discussions (FGDs) were conducted to explore fully socioeconomic, political, and cultural factors that might have had affected differences in *S. mansoni* prevalence between PHS and RES villages. Four trained research assistants were involved in conducting interviews and facilitating the FGDs.

#### Key informants’ interviews.

Thirty-nine KIIs were conducted with adult male and female key informants in eight villages. Same-gender researcher-administered interviews were conducted using an interview guide in Kiswahili which had open-ended questions and probes to allow for in-depth investigation of issues under investigation. Anonymity and confidentiality of the data gathered were ensured as there were no personal identifiers during data collection and on data collection forms. Data were documented by both taking notes and digital recordings. Permission to record the interview was sought before the interview sessions. Saturation was reached when there was no more new information coming from the informants.

#### Focus group discussions.

Sixteen FGDs further complemented interviews. Participants were selected conveniently with the help of village leaders. Participants came from among adult males and females who were not involved in the interviews. Discussions were held in primary schools and village premises separately for men’s and women’s groups. One pair of female and another pair of male research assistants facilitated the discussions as moderators and recorders, respectively, using an FGD guide in Kiswahili with the same questions as for interviews for the sake of methodological triangulation.

In most cases, the size of focus groups ranged from 6 to 9 participants, and the composition of focus groups took into consideration homogeneity (i.e., age-group, education, and socioeconomic status) and other related aspects emphasized in the FGD literature.^25,26^

Permission to record the discussions was sought before the sessions. Participants were encouraged to speak their views freely and spontaneously, and they were assigned numbers to use when contributing their views instead of names to ensure utmost anonymity and confidentiality. Discussions were held in natural settings outside in the schools’ and villages’ compounds under tree shades and quiet places. Saturation was reached when no more new information was coming out of the focus groups. The FGDs were documented by both taking notes and digital recordings.

### Data management and analysis.

An iterative approach was used in qualitative data collection and analysis. Digital-recorded interviews and discussions were typed, transcribed verbatim, and translated from Kiswahili to English. Data were summarized in matrices using variables of interest mentioned earlier. During preliminary analysis in the field, research topics on interview and FGD guides were used for initial categorization. Categories were inductively developed from data. The topics were then defined as codes (labels) and used to further code the data, and codes were developed manually by two investigators. This involved drawing up a list of coded categories and cutting and pasting each segment of transcribed data into one of these categories. This was easily performed manually as we had a small dataset as previously mentioned. Data were then reviewed several times for consistency and clarity. Constant comparative methods were performed by comparing raw data and summaries repeatedly. Quotes were taken where necessary, to support findings. Two investigators interpreted the data and assigned more or less the same meaning to the data. Validation and trustworthiness of the findings were established as analysis used explicit, systematic, and reproducible methods.^27^

### Ethical considerations.

Ethical approval for this study was provided by the Lake Zone Institutional Review Board of the National Institute for Medical Research. Study aims and objectives, procedures, potential risks, and benefits (full disclosure) were explained to participants through an information sheet read/provided to them. Adequate informed consent was sought through signature or thumbprint on the consent forms. Anonymity, confidentiality, and privacy were maintained, and participants were informed of their rights to refuse to take part in the study or to withdraw from the study at any time during the study without jeopardizing their rights to medical care.

In this study, consent was not regarded as “one off-event” but rather a process which was negotiated and renegotiated throughout the entire research process. In this article, the term informant is used to mean interviewee and the term participant is used to mean a person who participated in FGDs. Both are also referred to as study participants.

## RESULTS

### Sociodemographic characteristics of the study participants.

Distribution of KIs by village, *S. mansoni* status, gender, and category of informants is given in Table 1. The vast majority (18/20; 90% and 14/19; 74%) of the informants in both RES and PHS villages were males, respectively. Distribution of focus groups by village, *S. mansoni* status, gender, and number of informants is given in Table 2. In total, 16 FGDs were held with a total of 123 participants, 66 (33 males and 33 females) from RES villages and 57 (29 male and 28 female) from PHS villages (Table 2).

**Table 1:** Distribution of key informants by village, *Schistosoma mansoni* status, gender, and type of informants

Responding villages				Persistent hotspots			
	No. of interviews	Gender (M/F)	Type of informants		No. of interviews	Gender (M/F)	Type of informants
Kahumulo	3	M	Traditional leader	Bulima	5	M	Traditional leader
		M	Chairperson-school			M	Community health worker
		M	Muslim religious leader			M	Christian religious leader
Mhonze	7	M	Christian religious leader			F	Christian religious leader
		M	Christian religious leader			F	Christian religious leader
		M	Christian religious leader	Shinembo	4	M	Muslim religious leader
		M	Christian religious leader			M	Traditional leader
		M	Christian religious leader			F	Community health worker
		M	Traditional leader			M	Chairperson-school
		F	Assistant chairperson-school	Karumo	5	F	Christian religious leader
Shilingwa	5	M	Traditional leader			M	Christian religious leader
		M	Chairperson-school			M	Christian religious leader
		M	Chairperson-health			M	Chairperson-school
		M	Community health worker			M	Chairperson-health
		F	Christian religious leader	Luchili	5	M	Christian religious leader
Igalagalilo	5	M	Traditional leader			M	Christian religious leader
		M	Chairperson-health			F	Christian religious leader
		M	Chairperson-school			M	Chairperson-school
		M	Christian religious leader			M	Muslim religious leader
		M	Muslim religious leader				
Total	20			Total	19		

F = female; M = male.

**Table 2:** Distribution of focus groups by village, *Schistosoma mansoni* status, gender, and number of participants

Responding villages					Persistent hotspots				
	No. of sessions	No. of participants	No. of sessions	No. of participants		No. of sessions	No. of participants	No. of sessions	No. of participants
	Male	Male	Female	Female		Male	Male	Female	Female
Kahumulo	1	12	1	10	Bulima	1	8	1	9
Mhonze	1	6	1	8	Shinembo	1	7	1	6
Shilingwa	1	8	1	9	Karumo	1	7	1	7
Igalagalilo	1	7	1	6	Luchili	1	7	1	6
Total	4	33	4	33	Total	4	29	4	28

In Table 3, we summarize the sociodemographic characteristics of FGD participants in RES and PHS villages. Half of the FGD participants from RES villages were aged between 35 and 54 years. Slightly more than two-thirds of FGD participants from RES villages completed primary VII education, and a big proportion (39; 59%) of them were followers of the Catholic faith. Most of the participants were married, and a vast majority were engaged in subsistence farming. Slightly more than three-quarters of them belonged to Wasukuma, the dominant ethnic group in Mwanza region, and a quarter belonged to other ethnic groups. Adult male’s age in PHS villages ranged from 21 to 65 years. Females’ age ranged from 19 to 63 years. Slightly more than half were 35 years old. More importantly, slightly more than half completed primary VII education, and a big proportion (36; 63%) of them were followers of the Catholic faith. More than two-thirds of FGD participants were married. The vast majority were engaged in subsistence farming and belonged to the Wasukuma ethnic group in the Mwanza region (Table 3).

**Table 3:** Sociodemographic characteristics of focus group participants in RES and PHS villages

	RES villages			PHS villages		
	Male	Female	Total	Male	Female	Total
Age-groups (years)						
< 25	3	4	7	1	5	6
25–34	5	6	11	8	3	11
35–44	6	11	17	4	6	10
45–54	10	6	16	7	10	17
≥ 55	9	6	15	9	4	13
Education						
No formal education	4	7	11	4	13	17
Did not complete grade 7	5	3	8	3	2	5
Completed grade 4	23	21	44	17	13	30
Completed form 4	1	2	3	4	0	4
College education	0	0	0	1	0	1
Religion						
Catholics	16	23	39	15	21	36
Other Christians	10	9	19	11	6	17
Moslems	7	1	8	3	1	4
Marital status						
Married	30	21	51	25	14	39
Other relationship	3	12	15	4	13	17
Occupation						
Subsistence farmers	30	29	59	24	27	51
Fishing	2	0	2	0	0	0
Other	1	4	5	5	1	6
Ethnic groups						
Wasukuma	20	32	52	22	15	37
Other	13	1	14	7	13	20
	23	21	44	17	13	30

PHS = persistent hotspot; RES = responding.

### Perceptions on schistosomiasis as a priority health problem in the study villages.

Study participants in both RES and PHS villages mentioned several illnesses affecting people in their communities. However, most of them in both villages (RES and PHS) did not consider schistosomiasis as a priority health problem, unlike malaria and other acute conditions. The main reason being that malaria kills faster than schistosomiasis which takes a long time to kill. One FGD participant from one of the RES villages remarked:

If I were confronted with two disease conditions, I will start to treat malaria because it knocks you down very fast and once it catches a child, it is as if he/she has gone mad but schistosomiasis does not knock you down that fast and does not drive you mad. [RES-FFGD-2: Female FGD participant, Shilingwa village, Magu district]

This was also supported by an informant from PHS villages:

Schistosomiasis is a disease which is not given high priority in this village but malaria is given high priority although schistosomiasis is a very dangerous disease and affects almost everybody but this is due to lack of education on schistosomiasis. [HS-MKI-6: Male key informant, Shinembo village, Magu district]

### Village establishment and migration.

In both RES and PHS villages, it had been revealed that almost all villages were established long time ago but there had been reestablishment of villages during 1974 when Tanzania adopted the policy of “Ujamaa na kujitegemea” (socialism and self-reliance), during which Ujamaa villages (socialist villages) were established. Most of the people in both study villages were locals, with a few immigrants who were reported to have started moving in since the establishment and reestablishment of those villages for farming, fishing, and business purposes.

Both PHS and RES villages reported to having immigrants from more or less the same areas of the Lake Victoria and the western zone of Tanzania. Migrants were reported to have come from almost all districts of Mwanza region and other regions of the Lake and western zones. The only exception is that one RES village reported to have immigrants from neighboring East African countries of Rwanda and Burundi. One participant remarked:

There are so many migrants in our village for various reasons…some for agriculture, some for business or fishing. [PHS-MFG-5: Female participant, Karumo village, Sengerema.

### The leadership quality of villages and schools.

#### The village leadership.

Regarding village leadership style, interviews seemed to be more informative than focus groups. In all four RES villages, it was reported that village leadership was good and democratic in the sense that it allowed freedom of expression to prevail, and this gave leeway for village members to participate in different projects such as building shallow wells and latrines, and MDA as opposed to in all four PHS villages, whereby informants complained of leadership incompetence, autocracy, tribalism, favoritism, corruption, selfishness, and other malpractices including negligence. During one focus group, the issue of the autocracy of leadership came out as follows:

The leadership of this village is not good. It does not grant freedom of expression; it is dictatorial hence it is not progressive. Leaders use force (power) to govern…look you (SCORE project team) came here yesterday but the leadership did not inform the people that you were coming, had we been informed earlier, we would have attended in large numbers. [PHS-MFG-3: Male participant, Karumo village, Sengerema district]

The issue of negligence on the part of leadership in the same village is echoed in the following extract from one of the focus groups:

The weakness of this leadership is that they see people taking bath, swim, wash clothes and do other water contacts in the lake which is dangerous for them and public health but they don’t care. [PHS-FKI-1: Female informant, Bulima village, Busega district]

Regarding the leadership of another village, one informant lamented:

This leadership is not upright because it is less concerned with health issues for instance, there are no medicines in the dispensary, they don’t bother to see that people are educated on schistosomiasis, village revenue is misappropriated and leaders are the ones who benefit and do not implement what people want. [PHS-MKI-11: Male informant, Luchili village, Buchosa district]

More importantly, in RES villages, many study participants were of the view that leadership of their villages was good. The village development committees in collaboration with villagers were involved in MDA, and building of latrines, shallow wells, and classrooms in schools and villages. One key informant attested to the aforementioned assertion as follows:

The village and school leadership in this village is good as they work together in harmony. They inform parents about drug administration days, they boil drinking water for children, and they supervise drug administration and make sure that children get food before taking drugs. [RES-MKI-11: Male informant, Muhonze village]

#### The school leadership.

Regarding the school leadership style, the same pattern as that of village leadership emerged. Key informants’ interviews were more informative than FGDs. Findings revealed that generally in PHS villages, the school leadership style could be described as undemocratic and unable to create a conducive environment to facilitate children’s participatory teaching and learning atmosphere as opposed to RES villages. In PHS villages, it was reported that school leadership could not transform pupils to become action competent to combat schistosomiasis. They lacked transparency in implementing school projects, and some of them were afraid of convening meetings to deliberate on school developmental issues.

The following quote illustrates lack of ability on the part of school leadership to make school pupils’ action competent:

…At that school, children have not been prepared to become action competent as far as schistosomiasis infection is concerned like some pupils taking the lead to teach others. This is due to the deficiency of science teachers and the teachers who are there do not have adequate knowledge of schistosomiasis. This has made children ignorant of cause, symptoms and consequences of schistosomiasis. [PHS-MKI-3: Male informant, Bulima village, Busega district]

### Levels of involvement (participation) in health programs.

In both PHS and RES villages, it was evident that people were somehow involved in health programs in one way or another. However, the study revealed that there were little community participation and social engagement in schistosomiasis control programs in PHS than in RES villages. The reasons mentioned were mainly lack of understanding of schistosomiasis as infection and disease and poverty.

However, this study has also revealed that in both PHS and RES villages, there have been schistosomiasis control programs going on from time to time by the Ministry of Health and other development partners (including SCORE project). These have been mainly MDA for schistosomiasis and soil-transmitted helminths mostly in primary schools and to some extent in the villages. One informant from RES village said:

Village leaders mobilize people to keep the environment clean, with the emphasis on having and using clean and safe latrines, providing health education on schistosomiasis control for the entire community including treating the affected with PZQ and Albendazole. [RES-MKI-9: Male informant, Shilingwa village, Magu district]

On the other hand, it was reported that some people do not participate in schistosomiasis control programs because of ignorance and poverty. One informant remarked:

Many people in this village do not participate in health programs particularly schistosomiasis control because of lack of health education and poverty. They do not know how schistosomiasis is transmitted and prevented and cannot afford treatment because it is costly. [PHS-FKI-2: Female informant, Bulima village, Busega district]

### Motivation and commitment to participate in health programs on schistosomiasis.

In both RES and PHS villages, people were asked what motivates them to participate in schistosomiasis control programs and how committed they were in the said programs. Again, the same pattern emerged of many people who were motivated and committed among the RES compared with PHS villagers. One informant from a PHS village said:

People of this village seem not to be motivated and are not committed to control schistosomiasis as they have allocated themselves points (places) along the Lake Victoria shores (mialo) which they use for different water contacts activities for men and women separately. There is possibility of people to defecate and urinate in the lake during swimming and taking bath particularly school-aged children. Many people do not have latrines and their leaders seem to have sanctioned this arrangement as they are adamant on the issue...and the people are ignorant of how schistosomiasis is transmitted and treated as they resort to traditional medicines. [PHS-MKI-5: Male informant, Shinembo, Magu district]

### The vision of a healthy school and wider community.

Study participants were asked about their visions (wishes and hopes about health for future and what they considered important regarding health as far as schistosomiasis infection is concerned). There were no major differences between the views of RES and PHS villagers. Many participants were optimistic about the future. The optimists envisaged schools and wider communities free of schistosomiasis infection. However, there were a few pessimists who envisaged a bleak future as far as the schistosomiasis situation is concerned. There were three preconditions mentioned which if met will help the optimists to realize their dreams: basic education on schistosomiasis (e.g., cause, mode of transmission, symptoms, treatment, prevention, and control), regular MDA with PZQ, clean water supply and sanitation, and economic empowerment.

### The economy of RES and PHS villagers.

#### Main occupation.

Study participants in both RES and PHS villages almost unanimously pointed out subsistence farming as their main occupation. Other occupations mentioned were fishing, livestock-keeping, and petty businesses. It was reported that it was common for inhabitants of both RES and PHS villages to have more than one occupation (e.g., combining subsistence farming and livestock-keeping and fishing).

## DISCUSSION

Findings from our study revealed that in both PHS and RES villages, schistosomiasis was not given the priority unlike other diseases such as malaria. Schistosomiasis poses a public health challenge, but for several reasons, it is not necessarily considered a priority in national and local health policies and programs.^16,17^ However, other scholars have argued that control interventions in relation to schistosomiasis are more difficult when the disease is not perceived as a priority health problem, just like the case with our study villagers, and therefore risk factors are not considered as important.^28^

Human behavioral factors in disease causation in the study villages should not be viewed largely from a cultural ecological (micro-sociological) perspective. That is, the individual manifestations of culturally prescribed behavioral patterns are seen as risk factors for individual contraction of disease.^29^ The danger of viewing disease control intervention (in this case MDA) and human behavior at solely micro-sociological level is that individuals may be incorrectly considered responsible, even liable for their own disease persistence (i.e., victim-blaming). Even worse, entire PHS villagers, for instance, may be blamed for maintaining high *S. mansoni* prevalence.

To avoid such victim-blaming and truly understand disease causation, adoption of political–ecological (macro-sociological) perspective is crucial. From this perspective, disease is viewed at the level of the population, and *S. mansoni* prevalence in this case is seen as the result of sociopolitical and economic forces, operating through time and in some cases on a worldwide level. The macro-sociological perspective emphasizes larger social forces and not the cumulative effect of individual behaviors per se as the ultimate causes of poor health.^29^ Therefore, there is a dire need in the study villages to adopt a macro-sociological perspective in schistosomiasis transmission and control, as people’s health is not just an individual responsibility. Our health is, to a large extent, governed by the physical, social, cultural, and economic environment in which we live and work.^30^

There are specific cultural, religious, social, and economic factors which often account for not only the occurrence of schistosomiasis but also its transmission and control. Still, local people make judgments about what is in their economic interest, what should be regarded as clear or unclear, what is normal as opposed to abnormal behavior, and what is perceived to be a disease that requires action. These factors give insights into the way people seek diagnosis, cooperate with the physician, and adopt preventive measures against future illnesses.

The Ottawa Charter of Health Promotion emphasizes that promoting health is more than just providing health services. This is also true of the study villages. Peace, shelter, education, income, a stable ecosystem, sustainable resources, social justice, and equity are all necessary for achievement of health.^30^ Research has shown that living and working conditions, such as housing, income, social support, work stress, and education, make a difference in the number and quality of years lived.^31^ Furthermore, the Charter calls for people in the study villages to act as advocates for health through adopting “a population health” approach focusing on political, economic, social, cultural, environmental, behavioral, and biological factors, emphasizing the diverse determinants of health.^30^

The Charter through its five essential strategies, to build a healthy public policy, create supportive environments, strengthen community action, develop personal skills, and reorient health services,^30^ has a potential role to play in PHS villages to control schistosomiasis.

The WHO has defined health promotion as “the process of enabling people to increase control over, and to improve, their health.”^30^ The key word “enabling” underscores the need for a shift in power over health from bureaucracies to people, and recognizes that power and control are the central issues in health promotion, just as they are in Primary Health Care (PHC). This observation is true of the situation found in PHS villages. The definition also acknowledges the collective nature of health improvement and reinforces the argument presented earlier in this article that an individual notion of health is problematic because people cannot be seen in isolation. Individuals are embedded in systems that profoundly affect their behavior and their health. To improve health, communities need to gain more control over these systems through their full involvement in decision-making processes which is missing in PHS villages.

Much of the leadership style in PHS schools and villages is against the development of AC and health promotion ethos and resembles moralistic paradigm which is considered to totalitarian because it acts as a barrier toward realizing democratic schools and societies.^24^ Democratization, decentralization of power and resources, and commitment to human rights and social justice are the political dimensions of a supportive environment for health.^32,33^

There were drawbacks in governance, social engagement,^34^ motivation, and commitment in PHS schools and villages which tended to militate against schistosomiasis control in comparison to RES schools and villages. The reason being the totalitarian leadership style reported in PHS villages. Moreover, in PHS villages, it was also found that people were not action competent,^35^ were less committed, and hence not motivated to participate in schistosomiasis control programs compared with RES villages. Participation, as described variously elsewhere,^36–39^ is a major tenet of health promotion as encapsulated in the Charter.^30^ The fact that there had been very little community participation in schistosomiasis control in PHS villages calls for an urgent need to redress the situation through health promotion.^40^ Lack of community understanding of the importance of control measure (MDA in this case) and the role of the people in such measures seem to be outstanding in PHS villages.

Developing visions is another component of AC.^21^ In study villages, people developed visions of a healthy school and wider community in general, and how society and environment could be improved as far as schistosomiasis infection is concerned. This component dealt with the development of people’s ideas, dreams, and their perceptions about their future life and the society in which they live. It is about the conditions which one work with (i.e., high schistosomiasis prevalence) and would like to change how things will look like in the future. The fact that many people in the study villages were optimistic about the future situation of schistosomiasis is encouraging as having visions about the good life and future worlds is an important part of being action competent.^20–24^ However, in the study villages, people mentioned basic education on schistosomiasis, regular MDA with PZQ, and economic empowerment to be the three main prerequisites to realize their vision of schistosomiasis-free communities.

The main occupation of the people in the study villages (both PHS and RES) is subsistence farming. Just like in other parts of Tanzania where *S. mansoni* is endemic, the dynamics of socioeconomic and cultural aspects in schistosomiasis transmission and control should not be ignored.^41–43^ It has been argued that schistosomiasis is more prevalent than previously thought.^44^ Nevertheless, to our knowledge, this is the first qualitative study to address factors influencing village response to PZQ-based MDA in villages undergoing yearly treatments and achieving high coverage in Tanzania.

We conclude that the physical, social, economic, and political environments should be supportive to schistosomiasis control as it was the case with RES villages as opposed to PHS villages. A supportive environment is of paramount importance in health, as environment and health are interdependent and inseparable. The dimensions of supportive environment for health are the social, political, economic, and infrastructure.^33^

We also conclude that the meaning of PHS has been defined elsewhere.^45,46^

The present study aimed at identifying the factors contributing to sustaining these PHS villages which were found to be socioeconomic, cultural, and political in nature. Findings from this study have proven that in our endeavors to control schistosomiasis through MDA, we are missing socioeconomic, cultural, and political dimensions, which together with a positive infrastructure constitute dimensions of a supportive environment for health. Hence, we do concur with scholars who have suggested that for effective and sustainable schistosomiasis control through MDA, there is a need to adopt a biosocial approach at the local levels which in implementing PZQ-based MDA will take into consideration both biological and social determinants of transmission and control.^47–51^
